# A Case of West Nile Encephalitis That Develops into a Disease of Deep White Matter on MRI over a Two-Week Span

**DOI:** 10.1155/2016/4389691

**Published:** 2016-09-14

**Authors:** Adam Puchalski, Antonio Liu

**Affiliations:** White Memorial Medical Center, 1720 E. Cesar Chavez Ave., Los Angeles, CA 90033, USA

## Abstract

We present a case of serologically proved West Nile encephalitis. This patient had a normal MRI on admission. Given that the patient's clinical picture had worsened during her admission, a repeat MRI was performed, which demonstrated diffuse disease in the deep white matter of the brain. This is a case of West Nice encephalitis presenting as a disease of deep white matter that developed over a two-week span.

## 1. Introduction

West Nile is an arbovirus that belongs to the Flaviviridae family. It is spread by the* Culex* mosquitoes that have bitten infected animals. It has usually been found in Africa, Europe, the Middle East, and Asia. West Nile was first introduced in the United States in 1999. There was an outbreak of meningitis and encephalitis in the New York City borough of Queens [1]. The virus has spread and is found across the United States as well as Canada, Mexico, the Caribbean, and Latin America. There are between 1000 and 3000 cases per year in the United States, resulting in approximately 100–300 deaths [1].

## 2. Case Report

A 57-year-old Hispanic female presented with altered mental status, fever, and headache of 3 days' duration. Prior to that, patient was in her normal state of health; patient was able to ambulate on her own and able to perform all activities of daily living independently. The patient retuned from Mexico 3 days ago when she became ill and started experiencing her symptoms. She points to the back of her head and neck area when asked to locate her pain. The patient is in obvious distress due to the pain. The family notes that, when she is febrile, her mental status worsens. At the time of evaluation, she was oriented to person but not to time or place. She was lethargic. She denied any numbness, dizziness, weakness, neither seizures, nor changes in vision. She denied exposure to sick contacts, rhinorrhea, and itchy eyes and denied any mosquito bites.

**Figure 1:**
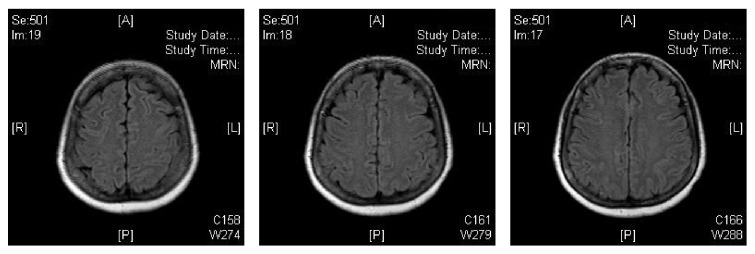
The patient's MRI on admission.

On neurologic exam, she was oriented to person but not to time or place. Patient was able to walk with no abnormalities in her gait. She was able to move all extremities with no sensory deficits. Cranial nerves 2–12 were formally tested and were intact. No visual defects were present. There was no pain present on knee extension when the patient's hip was flexed at 90 degrees. There was no flexion of the hips or knees present when the patient's head was lifted while she was lying supine. CT brain done on arrival revealed no evidence of acute intracranial pathology. MRI of the brain was performed and it revealed no evidence of acute infarct or any other intracranial pathology as demonstrated on Figure 1.

A lumbar puncture was performed. CSF studies showed a white blood cell count of 127 with 85% lymphocytes, 6 red blood cells, protein count of 177, and glucose of 54. Coccidioides antibody,* Cryptococcus* antigen, and VDRL were all negative. West Nile antibodies in the CSF were detected via ELISA and the results were as follows: IgM 6.10 (normal < 0.89) and IgG 2.08 (normal < 1.29).

**Figure 2:**
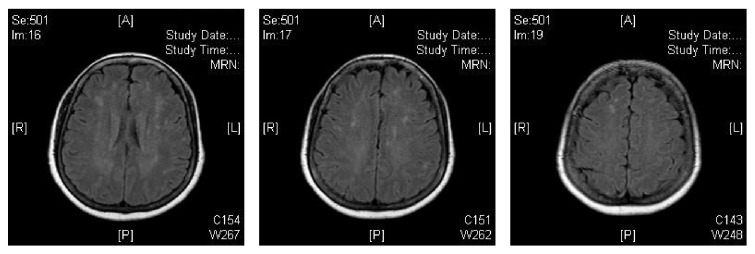
The patient's MRI performed two weeks later, which demonstrates white matter changes.

The patient was admitted and provided with supportive treatment. Her neurologic status slowly improved. Patient had EEG monitoring, which did not reveal any seizures. On the 16th day of her hospitalization, the patient's clinical picture worsened. Per nursing staff, patient had a facial droop and left sided weakness. On examination by the physician, facial droop was not noticed. She was following commands but quite lethargic. Patient was able to move both hands and able to move the toes on both feet. MRI was performed, which showed moderate periventricular white matter changes. This is demonstrated in Figure 2. The patient's clinical picture subsequently improved with continued supportive treatment. On the day of discharge, she was alert and oriented to person, place, and time. She was able to walk without any issues. She was able to follow commands and appropriately answer questions. She was subsequently discharged home.

## 3. Discussion

The majority of patients infected with West Nile are completely asymptomatic. Symptomatic patients present with flu like symptoms such as fatigue, maculopapular rash, headache, and gastrointestinal symptoms, which last for approximately one week. Others can have more severe symptoms, such as neuroinvasive disease. These include encephalitis or meningitis with accompanying dramatic motor paresis or paralysis. Currently, it is believed that 1 : 150 patients infected with West Nile will have such symptoms. These symptoms are more common among elderly, diabetic, and hypertensive patients as well as among patients with previous CNS insults [1].

Neuroimaging is frequently normal, with normal MRI findings in up to 30% of cases. Guth et al. presented two cases with MRI findings that demonstrated involvement in the thalamic nuclei down to the spinal cord [2]. Rosas and Wippold II presented a serologically proven case of West Nile encephalitis that demonstrated disease in the basal ganglia and in the thalami [3].

Our case demonstrates the rapid evolution of West Nile encephalitis. This is the first case report where the patient had a normal MRI on admission. In a span of approximately two weeks, her repeat MRI was vastly different, showing deep white matter disease in the periventricular area.

Ali et al. demonstrated an important trend for improved outcomes in patients without parenchymal or meningeal abnormalities on FLAIR imaging or T2WI. Additionally, patients with increased signal intensity in the brain and brainstem on FLAIR imaging and T2WI had the worst outcomes. Meningeal involvement was also mentioned and those patients suffered from severe residual neurologic deficit [4].

Imaging findings throughout the literature have demonstrated that West Nile can affect many parts of the brain, with no specific area being favored. Both the CDC and the Louisiana State University Health Science Centers have established an MRI registry that is specifically devoted to collecting images from West Nile cases. Because of the growing epidemic, this database can prove to be a tremendous learning tool for providers. MRI recognition of West Nile virus can not only aid in definitive diagnosis but can also provide prognostic information.
